# Sterol flux rewiring: Cholesterol biosynthesis as a dynamic signaling network

**DOI:** 10.1016/j.jbc.2026.113381

**Published:** 2026-07-30

**Authors:** Marc Poirot, Philippe de Médina, Sandrine Silvente-Poirot

**Affiliations:** Team INOV, Cancer Research Center of Toulouse, Inserm, CNRS, University of Toulouse, Toulouse, France

**Keywords:** cholesterol biosynthesis, sterol metabolism, sterol flux rewiring, oxysterols, EChA, cancer metabolism

## Abstract

Cholesterol biosynthesis is among the best-characterized metabolic pathways in biology, yet a fundamental question remains unresolved: Why does this pathway generate more than twenty enzymatic reactions and numerous structurally distinct intermediates if cholesterol is its major biological end product? Over the past several decades, biochemical, genetic, pharmacological, biophysical, and lipidomic studies have progressively revealed that many sterol intermediates are not merely transient precursors. Instead, they possess distinct biophysical, signaling, and oxidative properties that contribute directly to cellular physiology and disease. However, these discoveries have largely been interpreted within separate biological and experimental contexts, including inherited disorders of cholesterol biosynthesis, membrane biology, nuclear receptor signaling, oxysterol metabolism, and pharmacological inhibition of distal sterol enzymes. Here, we propose that sterol flux rewiring provides an integrative framework that connects these independent observations into a unified view of cholesterol metabolism. In this framework, biological responses emerge from dynamic redistribution of metabolic flux, generating distinct sterol states characterized by specific membrane properties, signaling activities, oxidative potentials, and downstream metabolic outputs rather than by the accumulation of individual metabolites alone. This perspective explains how changes in sterol composition reshape membrane organization, oxidative diversification, and interconnected signaling networks, including the epoxycholestanoid pathway. It also provides a coherent framework for understanding how alterations in cholesterol metabolism contribute to development, immunity, neurobiology, ageing, regeneration, and cancer, while highlighting new opportunities for therapeutic strategies aimed at reprogramming sterol-state organization rather than simply inhibiting cholesterol synthesis.

Cholesterol occupies a unique position in biology. Since Michel-Eugène Chevreul first isolated and named the unsaponifiable lipid *cholestérine* in (1, 2), it has become one of the most intensively studied molecules in biochemistry because of its essential roles in membrane organization, steroid hormone biosynthesis, bile acid production, lipoprotein metabolism, and cellular homeostasis. Over the past two centuries, biochemical, genetic, and physiological studies have established cholesterol metabolism as one of the best-characterized metabolic systems in biology. Yet its organization is still interpreted largely through the production and abundance of cholesterol itself.

This cholesterol-centered view was profoundly shaped by the pioneering work of Konrad Bloch, who established cholesterol biosynthesis as a highly ordered sequence of enzymatic reactions (3) and proposed that successive transformations progressively optimized sterol structure for membrane function (4). Subsequent discoveries, including the identification of SREBP-dependent transcriptional regulation, intracellular cholesterol trafficking, and the molecular basis of inherited disorders of cholesterol biosynthesis, greatly expanded this framework. Even so, most biosynthetic intermediates continued to be regarded primarily as transient precursors whose biological significance derived from their eventual conversion into cholesterol.

Yet observations accumulated over several decades are difficult to reconcile with this classical interpretation. Studies spanning membrane biophysics, developmental genetics, pharmacology, nuclear receptor biology, and oxidative sterol metabolism have shown that individual sterol intermediates possess physicochemical and biological properties that are not shared by cholesterol. Beyond serving as biosynthetic intermediates, post-lanosterol sterols regulate membrane organization, nuclear receptor signaling, redox homeostasis, cell proliferation, immune responses, and tissue regeneration. Oxidative metabolism further expands this functional diversity through the generation of oxysterols and epoxycholestanoids (EChAs) with distinct signaling activities (5, 6, 7).

None of these observations are new. For decades, biochemical and physiological studies have investigated the membrane properties, metabolic transformations, and regulatory activities of individual biosynthetic and oxidized sterols (4, 8, 9, 10). What has remained largely missing is an integrative framework capable of connecting these findings across human genetics, membrane biology, pharmacology, redox metabolism, and cell signaling.

In this Review, we propose sterol flux rewiring as such a framework. The term describes the redistribution of metabolic flux across post-lanosterol biosynthetic and oxidative pathways, generating characteristic sterol compositions with distinct biophysical, signaling, and metabolic properties. From this perspective, genetic variation, differentiation, oxidative stress, physiological adaptation, and pharmacological intervention do not simply alter the rate of cholesterol synthesis. They reshape the relative abundance, metabolic fate, and biological interactions of interconnected sterol intermediates.

This framework extends rather than replaces the classical model of cholesterol biosynthesis. Cholesterol remains the principal structural sterol and a central regulator of cellular homeostasis, but it is produced within a broader metabolic network whose intermediates and downstream metabolites also contribute to cellular function. Sterol flux rewiring therefore provides a common conceptual framework for integrating observations that have often been investigated independently, without implying that the biological importance of individual sterols has only recently been recognized. If cholesterol biosynthesis produces so many structurally distinct intermediates, why should cholesterol alone be considered biologically relevant?

The following sections develop this concept from three complementary perspectives. We first examine how post-lanosterol biosynthesis generates distinct sterol profiles and how these intermediates influence membrane organization, receptor signaling, homeostatic regulation, and oxidative potential. We then explore how oxidative diversification expands this organization through oxysterols and the EChA network. Finally, we discuss how sterol flux rewiring contributes to cancer, immunity, neurobiology, ageing, and regeneration, and consider its methodological and therapeutic implications.

## Architecture of cholesterol biosynthesis

### From linear pathway to functional organization

Cholesterol biosynthesis is traditionally depicted as a linear sequence of enzymatic reactions leading from acetyl-CoA to cholesterol. Although this representation accurately describes the order of biochemical transformations, it captures only part of the functional organization of sterol metabolism. The conversion of lanosterol into cholesterol requires multiple demethylation, reduction and isomerization reactions that generate structurally distinct sterol intermediates (11). As discussed above, Bloch proposed that this complexity reflects progressive evolutionary optimization of sterol structure rather than unnecessary biochemical redundancy (4).

The mevalonate pathway provides the first indication of this organization. Acetyl-CoA is converted into mevalonate through HMG-CoA reductase (HMGCR), the rate-limiting enzyme of cholesterol biosynthesis. HMGCR is regulated by multiple feedback mechanisms. Early studies by Siperstein demonstrated feedback inhibition of cholesterol synthesis (12), whereas subsequent work identified the SREBP-SCAP-INSIG system as the central regulatory axis controlling sterol homeostasis (13, 14). Importantly, the mevalonate pathway extends well beyond cholesterol synthesis. Isoprenoid intermediates, including isopentenyl pyrophosphate, geranyl pyrophosphate and farnesyl pyrophosphate (FPP), serve as precursors for protein prenylation, ubiquinone biosynthesis and dolichol metabolism (13, 15). Changes in mevalonate metabolism therefore influence numerous cellular processes independent of cholesterol production.

The first major metabolic branch occurs at FPP, where flux is partitioned between sterol and non-sterol pathways. Sterol synthesis then proceeds through squalene and lanosterol, the first cyclic sterol intermediate. Beyond lanosterol, biosynthesis follows the partially overlapping Bloch and Kandutsch–Russell pathways, whose relative contributions vary among tissues and physiological conditions (3, 11, 16).

This organization explains why genetic defects or pharmacological inhibition of individual post-lanosterol enzymes frequently produce phenotypes that cannot be predicted from cholesterol concentrations alone. Perturbing distal reactions reshapes intermediate sterol pools, modifies membrane properties, and alters the availability of substrates for oxidative metabolism. Cellular responses therefore reflect not only cholesterol abundance but also the composition of the surrounding sterol environment.

**Figure 1:**
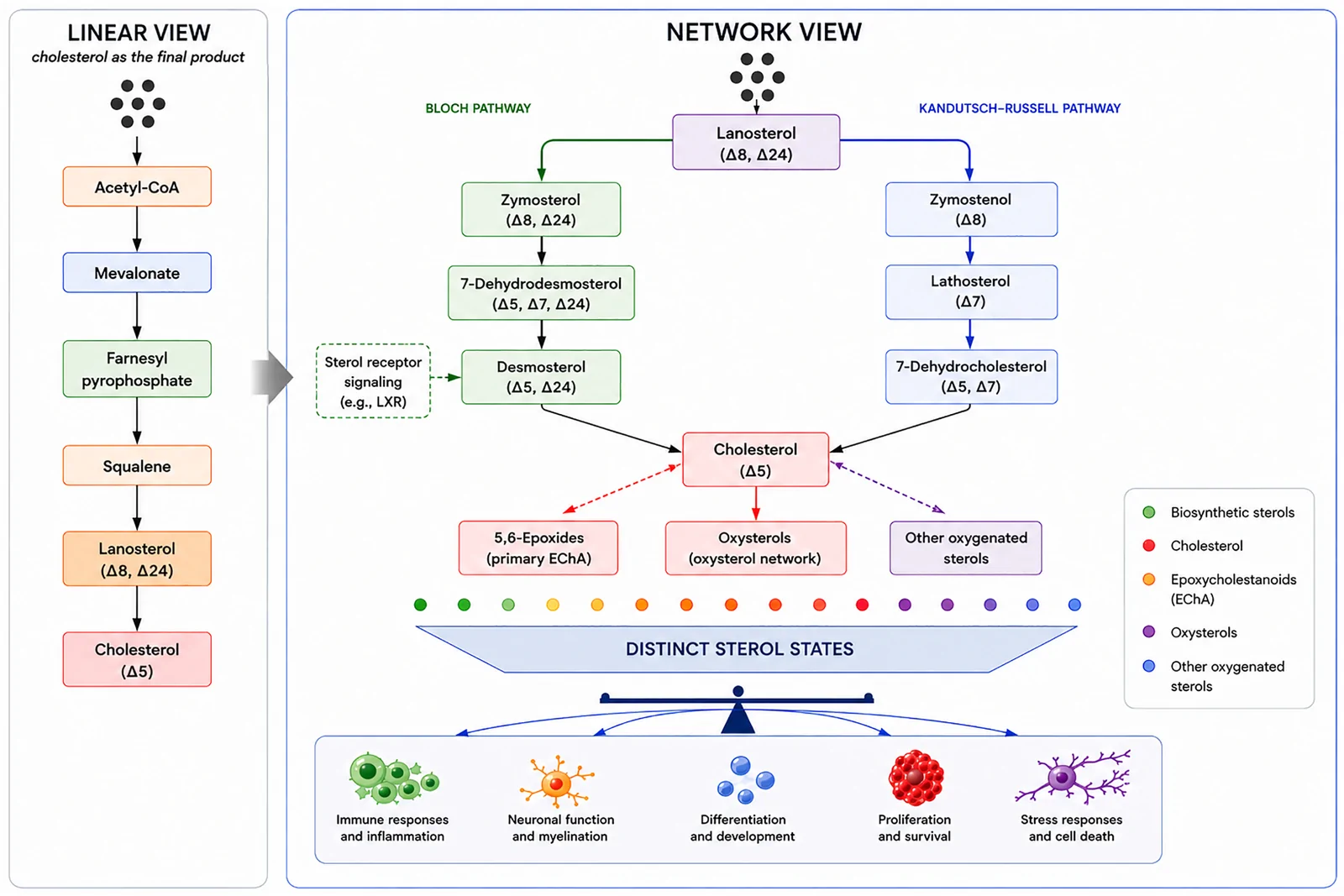
**Sterol flux rewiring transforms cholesterol biosynthesis from a linear pathway into a distributed metabolic signaling network.** The classical representation of cholesterol biosynthesis depicts a linear sequence of enzymatic reactions culminating in cholesterol production. In contrast, the network view emphasizes that metabolic flux is distributed across interconnected post-lanosterol pathways, including the Bloch and Kandutsch-Russell branches, generating sterol intermediates with distinct biochemical and signaling properties. Redistribution of sterol flux alters the relative abundance of biosynthetic sterols, oxysterols, epoxycholestanoids (EChA) and other oxygenated sterols, generating distinct sterol states with characteristic signaling properties. Consequently, relatively modest changes in sterol composition can reshape membrane organization, oxidative metabolism and sterol-dependent signaling, producing disproportionate effects on immune regulation, neuronal function, differentiation, proliferation, and stress adaptation. Rather than cholesterol abundance alone, the relative organization of biosynthetic sterols and their oxygenated derivatives define sterol states that influence cellular physiology. The colored circles schematically illustrate the relative contribution of biosynthetic sterols, oxysterols, epoxycholestanoids, and other oxygenated sterols to different sterol states.

Viewed from this perspective, cholesterol biosynthesis is not simply a linear pathway but a dynamic metabolic network capable of generating distinct sterol states according to enzymatic activity, cellular context and metabolic demand. These sterol states provide the conceptual framework for the regulatory functions of biosynthetic sterols discussed in the following sections (Fig. 1).

### Post-lanosterol metabolism generates distinct sterol profiles

The post-lanosterol phase of cholesterol biosynthesis comprises a series of demethylation, reduction and isomerization reactions that convert lanosterol into cholesterol. For many years, these reactions were regarded simply as the final biosynthetic steps required to produce cholesterol, whereas the intermediate sterols were viewed as transient metabolites with little biological significance. This interpretation changed fundamentally through the study of inherited disorders of cholesterol biosynthesis.

Sterol profiling revealed that these disorders are characterized not only by reduced cholesterol synthesis but also by the accumulation of specific intermediates immediately upstream of the defective enzyme (17, 18). Consequently, mutations affecting different post-lanosterol enzymes generate distinct sterol compositions associated with characteristic developmental and metabolic phenotypes (19).

Smith-Lemli-Opitz syndrome (SLOS), caused by mutations in DHCR7, provides one of the clearest examples. In addition to reduced cholesterol synthesis, affected individuals accumulate large amounts of 7-DHC, a sterol with physicochemical and metabolic properties that differ markedly from those of cholesterol (20). Likewise, mutations in SC5D cause lathosterolosis with lathosterol accumulation, whereas DHCR24 deficiency leads to desmosterolosis characterized by elevated desmosterol levels (21, 22). Although these disorders all impair the same biosynthetic pathway, they produce distinct clinical phenotypes that cannot be explained by cholesterol deficiency alone.

These observations established an important principle: the identity of the accumulating sterol intermediate matters. Even closely related sterols differ in membrane behavior, protein interactions, susceptibility to oxidation and signaling capacity (4, 19, 23, 24, 25, 26). Distal cholesterol biosynthesis therefore generates a series of chemically and biologically distinct sterol intermediates rather than interchangeable precursors awaiting conversion into cholesterol.

Human genetics thus provided the first compelling evidence that post-lanosterol enzymes do more than control cholesterol production. By determining which intermediates accumulate, they define characteristic sterol profiles with distinct functional properties. This concept forms the basis for the pharmacological studies discussed in the following section and, more broadly, for the notion that redistribution of sterol flux generates biologically distinct sterol states.

### Pharmacological perturbations reveal redistribution of sterol flux

Human genetics demonstrated that disruption of individual post-lanosterol enzymes generates characteristic sterol profiles. Pharmacological intervention provides a complementary approach by showing that these profiles can be remodeled in a controlled, reversible and graded manner. Rather than simply suppressing cholesterol synthesis, inhibition of distal biosynthetic enzymes redistributes metabolic flux, generating alternative sterol compositions with distinct biological consequences.

Early studies showed that inhibitors acting at different positions within the distal pathway consistently induce the accumulation of sterol intermediates immediately upstream of the inhibited reaction. Initially regarded as biochemical markers of enzyme inhibition, these metabolites were subsequently recognized as biologically relevant components of the cellular sterol environment. A pivotal series of studies by Lasunción and colleagues demonstrated that pharmacological inhibition at different steps of post-lanosterol biosynthesis differentially regulates cell cycle progression and proliferation in human cancer cells (27, 28, 29). These findings provided some of the first direct evidence that altering sterol composition, rather than cholesterol abundance alone, can modify cellular phenotypes.

Subsequent studies considerably broadened this concept. Numerous clinically approved drugs developed for unrelated indications were found to interfere with distal cholesterol biosynthesis by targeting enzymes such as DHCR7, EBP, or DHCR24 (30, 31, 32, 33). Because each inhibitor perturbs a different regulatory node within the pathway, individual compounds generate characteristic sterol profiles that reflect both their molecular targets and their pharmacological selectivity. Consequently, drugs producing comparable reductions in cholesterol synthesis may nevertheless trigger markedly different biological responses by redistributing sterol flux toward distinct sterol states.

Unlike inherited enzyme deficiencies, pharmacological inhibition is usually partial and frequently affects more than one target. The resulting sterol composition therefore reflects the integrated effects of graded inhibition across an interconnected metabolic network rather than complete blockade of a single enzymatic reaction. Pharmacological intervention thus provides access to a continuum of sterol states that complement the discrete sterol profiles observed in monogenic disorders.

These experimental models also revealed that the consequences of sterol redistribution extend well beyond cholesterol biosynthesis itself. Altering post-lanosterol metabolism modifies membrane organization, changes the availability of substrates for oxidative metabolism and influences multiple sterol-dependent signaling pathways. Pharmacological perturbation therefore represents more than a means of inhibiting enzyme activity; it offers a powerful experimental strategy for dissecting the functional organization of sterol metabolism.

This perspective has important conceptual implications. Distal cholesterol biosynthetic enzymes are not simply sequential catalysts but regulatory nodes whose activity determines the organization of the intracellular sterol organization. Pharmacological inhibitors should therefore be viewed not only as enzyme antagonists but also as tools that deliberately redirect sterol flux toward defined sterol states. The following sections illustrate how different classes of compounds exploit this property to reveal the remarkable plasticity of sterol metabolism.

### AEBS ligands reveal the plasticity of post-lanosterol sterol metabolism

Among the pharmacological models that have shaped our understanding of cholesterol biosynthesis, ligands of the antiestrogen-binding site (AEBS) provide one of the clearest demonstrations that biological responses depend on the redistribution of sterol flux rather than on cholesterol depletion itself. Unlike conventional enzyme inhibitors, these compounds remodel post-lanosterol metabolism in a ligand-dependent manner, generating characteristic sterol states whose composition reflects both the molecular target and the chemical structure of the ligand (34, 35).

The first evidence that pharmacological perturbation of distal cholesterol biosynthesis could profoundly alter sterol composition came from triparanol (MER-29), the hypocholesterolemic drug withdrawn because of its severe toxicity. By blocking the final steps of cholesterol biosynthesis, triparanol induced the accumulation of precursor sterols instead of producing uniform cholesterol depletion (36, 37). Similar observations were subsequently made with structurally unrelated compounds, including triphenylethylene derivatives. Tamoxifen, for example, was found to promote the accumulation of post-lanosterol sterol intermediates independently of estrogen receptor signaling, suggesting the existence of intracellular pharmacological targets directly connected to sterol metabolism (38, 39).

The molecular basis of these observations became clearer with the characterization of the AEBS. Initial studies identified emopamil-binding protein (EBP), the Δ8–Δ7 sterol isomerase, as one component of the binding site (40, 41, 42). However, recombinant EBP alone failed to reproduce the high-affinity pharmacology observed in mammalian cells. Kedjouar and colleagues subsequently demonstrated that high-affinity AEBS requires the association of EBP with DHCR7, revealing a multiprotein complex embedded within the distal cholesterol biosynthetic pathway (30).

This work also uncovered a previously unrecognized property of sterol metabolism. Structurally related AEBS ligands generated markedly different sterol profiles despite interacting with the same molecular complex. Non-phenolic triphenylethylenes such as tamoxifen and clomiphene predominantly promoted the accumulation of zymostenol, consistent with preferential inhibition of EBP, whereas phenolic derivatives including 4-hydroxytamoxifen and raloxifene favored the accumulation of Δ24 sterols, indicating greater inhibition of DHCR24. Diphenylmethane derivatives such as PBPE and tesmilifene produced broader sterol profiles involving both pathways (30). These observations demonstrated that the biological consequences of pharmacological intervention are determined not simply by target engagement but by the way each ligand redistributes sterol flux through an interconnected metabolic network.

Structural studies subsequently provided a mechanistic explanation for these findings. The crystal structure of human EBP in complex with tamoxifen revealed a hydrophobic ligand-binding cavity within the transmembrane domain (43). Molecular docking strongly suggested that cholesterol 5,6-epoxides (5,6-ECs) occupy the same, or an overlapping, binding pocket (44). Together with kinetic analyses showing non-competitive inhibition of EBP by tamoxifen (30, 42), these observations indicate that ligand binding and sterol isomerization are functionally coupled without sharing the catalytic site. This organization offers a structural basis for understanding how chemically related compounds differentially redirect sterol metabolism.

The physiological significance of these discoveries increased further when the EBP-DHCR7 complex was identified as cholesterol epoxide hydrolase (ChEH), directly linking post-lanosterol biosynthesis to 5,6-EC metabolism (45). Several AEBS ligands increase cholesterol epoxidation and promote the accumulation of 5,6-ECs, thereby coupling biosynthetic and oxidative sterol metabolism (46). Importantly, this response also depends on the cellular redox environment. Whereas tamoxifen enhances cholesterol epoxidation, the endogenous ChEH inhibitor dendrogenin A (DDA) induces catalase expression and limits cholesterol epoxidation despite acting on the same enzymatic complex (47, 48). These observations illustrate that the biological consequences of ChEH modulation emerge from the combined regulation of sterol flux and cellular redox homeostasis.

AEBS ligands therefore represent far more than pharmacological inhibitors of distal cholesterol biosynthesis. They established that relatively small changes in ligand structure can redirect sterol flux toward distinct sterol states, thereby reshaping membrane composition, oxidative metabolism and downstream signaling with only limited effects on total cholesterol. More broadly, they provided one of the first experimental demonstrations that cholesterol biosynthesis behaves as a highly adaptable metabolic network rather than as a rigid linear pathway.

### U18666A reveals the interconnected nature of sterol metabolism

Among the pharmacological probes used to investigate sterol metabolism, U18666A provides one of the clearest demonstrations that cholesterol homeostasis emerges from the integration of multiple interconnected processes rather than from the activity of individual enzymes or transporters. Unlike AEBS ligands, which primarily remodel post-lanosterol biosynthesis, U18666A simultaneously perturbs intracellular cholesterol trafficking, distal sterol biosynthesis and cholesterol epoxide metabolism, thereby exposing the functional connectivity of the sterol network (49).

U18666A was originally identified through its ability to induce lysosomal cholesterol accumulation and rapidly became the reference pharmacological model of Niemann–Pick type C (NPC) disease (50). Early studies showed that the compound blocks the export of LDL-derived cholesterol from late endosomes and lysosomes, resulting in impaired cholesterol esterification and altered sterol-dependent feedback regulation. The subsequent identification of NPC1 as the principal lysosomal cholesterol transporter, together with the demonstration that U18666A binds directly to NPC1, established the molecular basis of this NPC-like phenotype (51, 52, 53). For many years, U18666A was therefore viewed primarily as an inhibitor of intracellular cholesterol trafficking.

This interpretation changed when sterolomic analyses revealed that U18666A also remodels post-lanosterol cholesterol biosynthesis. Accumulation of desmosterol and other precursor sterols demonstrated that its effects extend well beyond cholesterol transport. Biochemical studies identified DHCR24 as one pharmacological target, whereas more recent cryo-electron microscopy analyses showed that U18666A also binds within the sterol-binding cavity of EBP (43, 54, 55). U18666A therefore acts simultaneously on several key components of sterol metabolism rather than on a single cellular process.

This multi-target organization has important mechanistic implications. Because EBP forms cholesterol epoxide hydrolase (ChEH) together with DHCR7 (45), perturbation of EBP is expected to influence not only post-lanosterol biosynthesis but also 5,6-EC metabolism. U18666A is therefore likely to remodel intracellular cholesterol trafficking, sterol composition and oxidative sterol metabolism simultaneously, functionally linking metabolic modules that have traditionally been investigated independently.

The temporal organization of these events, however, remains largely unresolved. Although U18666A directly interacts with NPC1, DHCR24 and EBP, it is still unknown whether defects in cholesterol trafficking precede changes in sterol biosynthesis or whether altered sterol composition subsequently amplifies trafficking defects through secondary feedback mechanisms. Resolving this sequence of events will be important for understanding how perturbations propagate through the sterol network.

Recent observations further support this integrated view. A non-hydrolysable analogue of cholestane-3β,5α,6β-triol-3β sulfate (CDSN) inhibits the uptake of cholesterol, CT and 5,6-ECs while inducing giant multilamellar bodies that closely resemble those observed in NPC cells, consistent with impaired NPC1-dependent sterol transport (56). Although the molecular mechanism remains to be established, these findings raise the intriguing possibility that metabolites generated downstream of 5,6-EC metabolism participate in the regulation of intracellular sterol trafficking.

Rather than acting as a selective inhibitor of cholesterol transport, U18666A therefore illustrates how perturbation of a single pharmacological node propagates across multiple interconnected modules of sterol metabolism. Cholesterol trafficking, post-lanosterol biosynthesis and oxidative sterol metabolism cannot be considered independently but function as components of an integrated metabolic network. This systems-level organization provides the conceptual bridge between the pharmacological evidence presented in this chapter and the regulatory functions of sterol intermediates discussed in the following sections.

### Human sterol profiles reflect dynamic sterol states

Experimental studies demonstrate that genetic and pharmacological perturbations redistribute sterol metabolism into distinct biochemical states. Human sterolomic studies indicate that this metabolic diversity is not restricted to experimental models but represents an intrinsic property of human physiology. Rather than operating through a single invariant biosynthetic program, human tissues exhibit substantial variability in post-lanosterol sterol composition despite maintaining cholesterol homeostasis.

For many years, sterol metabolism was assessed primarily by measuring plasma cholesterol and lipoproteins. Although clinically invaluable, these parameters provide only limited information about the organization of the biosynthetic network itself. The development of GC-MS and LC-MS transformed this field by enabling the simultaneous quantification of multiple non-cholesterol sterols (57, 58, 59, 60). These analytical approaches revealed that circulating precursor sterols are sensitive indicators of pathway activity and provide metabolic information inaccessible through cholesterol measurements alone.

One of the most important observations emerging from these studies is that individuals with similar plasma cholesterol concentrations often display markedly different precursor sterol profiles (57, 58, 59). Likewise, comparable rates of cholesterol synthesis may be associated with distinct distributions of post-lanosterol intermediates, indicating that cholesterol homeostasis can be maintained through different biosynthetic organizations. Stable-isotope tracing reached the same conclusion by demonstrating that different tissues preferentially utilize distinct combinations of Bloch and Kandutsch–Russell reactions while producing the same final product, cholesterol (61). Cholesterol homeostasis can therefore be maintained through multiple biosynthetic organizations, each characterized by a distinct sterol composition.

Together, these observations demonstrate that sterol metabolism operates through multiple physiological sterol states rather than a single metabolic equilibrium.

Interindividual differences arise from the combined influence of genetic background, age, sex, endocrine status, nutrition, metabolic disease, and pharmacological exposure (32, 57, 59, 62). Human sterol profiles should therefore be viewed as dynamic metabolic phenotypes that continuously adapt to physiological and pathological conditions rather than as fixed biochemical characteristics.

These observations complete an important progression developed throughout this chapter. Human genetics demonstrated that disruption of individual enzymes generates characteristic sterol profiles. Pharmacological studies showed that these profiles can be remodeled experimentally. Human sterolomics now establish that analogous sterol states occur naturally under physiological conditions. Together, these complementary approaches provide the experimental foundation for the central concept developed in this Review: cholesterol metabolism operates as a dynamic network capable of generating multiple biologically distinct sterol states.

The existence of distinct sterol states raises an important question: How do these alternative sterol compositions translate into biological function? The following sections examine how receptor recognition and oxidative metabolism transform metabolic diversity into signaling diversity.

## Sterol intermediates as regulatory metabolites

The preceding sections establish that redistribution of sterol flux generates distinct sterol states characterized by specific post-lanosterol sterol compositions. The next question is whether these sterol profiles simply reflect the organization of cholesterol biosynthesis or actively shape cellular physiology.

Evidence accumulated over several decades strongly supports the latter interpretation. Biosynthetic sterol intermediates are not merely transient molecules awaiting conversion into cholesterol. Many possess intrinsic physicochemical and regulatory properties that influence membrane organization, protein function, intracellular trafficking, transcriptional programs and oxidative metabolism (4, 19, 63). Their biological relevance is therefore not a recent discovery. Rather, what has progressively emerged is the realization that observations originating from membrane biophysics, human genetics, pharmacology and redox biology can be integrated within a common conceptual framework.

These properties arise from remarkably subtle structural modifications introduced during sterol biosynthesis. Variations in double-bond position, side-chain saturation or sterol isomerization are sufficient to alter membrane behavior, molecular recognition, receptor activation and metabolic reactivity (4, 11). Consequently, sterols occupying adjacent positions within the biosynthetic pathway cannot be regarded as functionally interchangeable despite their close structural similarity.

**Figure 2:**
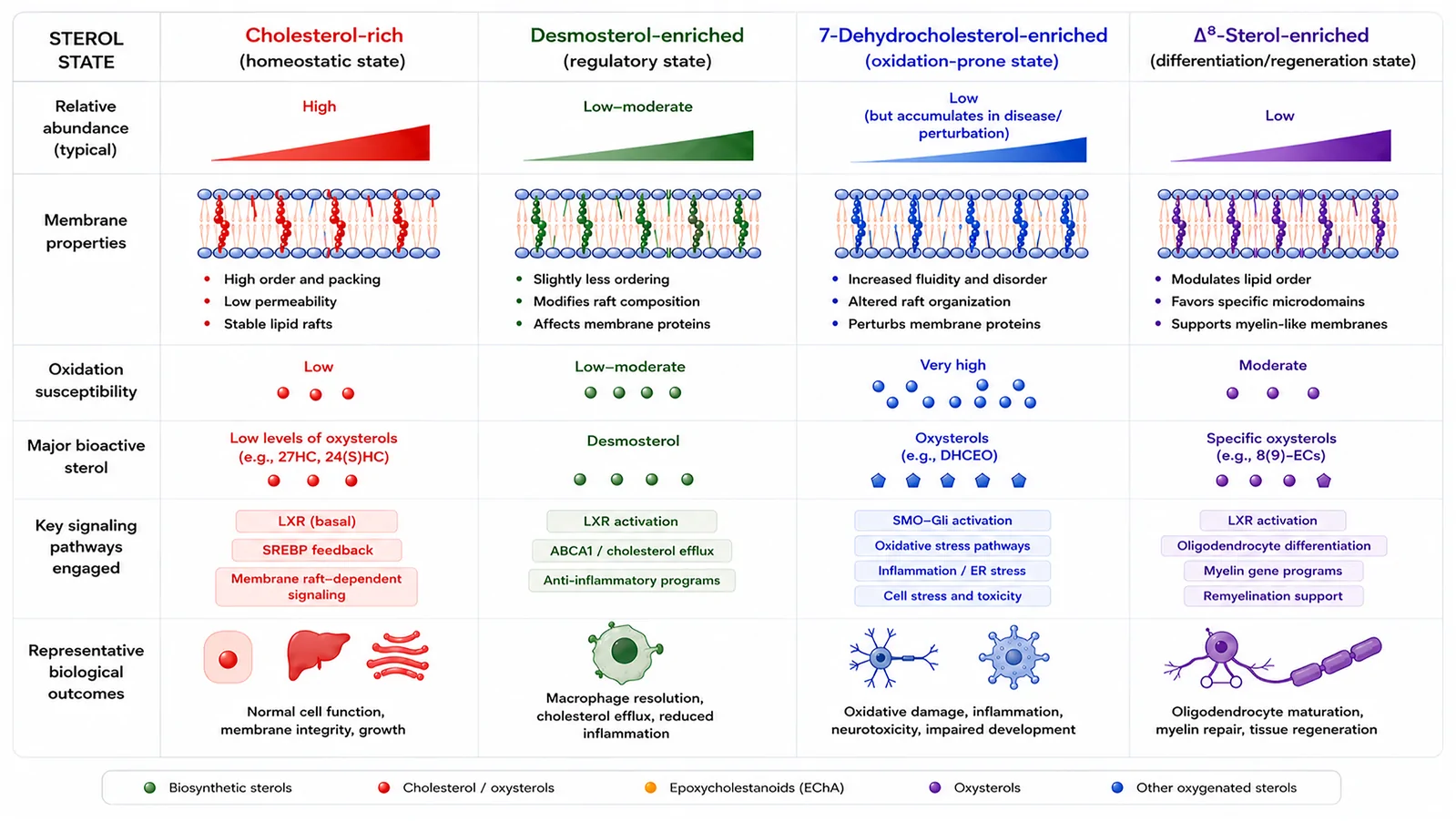
**Distinct sterol states generate distinct membrane, signaling and oxidative landscapes.** Changes in post-lanosterol sterol composition generate distinct sterol states, each characterized by a specific combination of membrane properties, signaling activities, and susceptibility to oxidative diversification. Cholesterol-rich membranes represent the canonical homeostatic state, whereas accumulation of specific biosynthetic intermediates generates alternative functional states. Desmosterol promotes LXR-dependent transcriptional programs associated with cholesterol efflux and resolution of inflammation. Accumulation of 7-dehydrocholesterol creates an oxidation-prone state characterized by increased formation of bioactive oxidation products such as DHCEO and activation of Smoothened (SMO)-Gli signaling, thereby promoting oxidative stress and neurotoxicity. In contrast, accumulation of Δ^8^-sterols has been associated with differentiation and remyelination programs. Together, these examples illustrate how changes in sterol composition remodel membrane organization, signaling pathways and redox biology, thereby generating distinct functional sterol states that influence cell physiology.

The following sections examine how this progressive structural diversification is translated into cellular function. Using representative examples, we illustrate how biosynthetic sterols regulate membrane organization, receptor signaling, metabolic homeostasis and oxidative diversification. Together, these examples show that the biological consequences of sterol flux rewiring arise not simply from changes in cholesterol abundance, but from the functional properties of the sterol intermediates that define each sterol state (Fig. 2).

### Structural diversity as a source of functional diversity

The functional diversity of biosynthetic sterol intermediates originates from the progressive structural modifications introduced during cholesterol biosynthesis. More than twenty enzymatic reactions sequentially remodel the sterol nucleus and side chain through demethylation, reduction and double-bond isomerization (11). The remarkable evolutionary conservation of this biosynthetic sequence suggests that these intermediates contribute functions extending beyond their role as transient precursors of cholesterol.

Konrad Bloch was the first to formalize this idea by proposing that cholesterol biosynthesis represents a process of progressive molecular refinement in which successive intermediates incrementally improve sterol performance within biological membranes (4). Subsequent work has considerably broadened this concept. Structural modifications introduced during biosynthesis influence not only membrane behavior but also protein recognition, metabolic reactivity and susceptibility to oxidative transformation.

The first evidence came from membrane biophysics. Replacing cholesterol with closely related precursor sterols alters membrane order, permeability and lipid-domain organization (24, 25, 64, 65). Because membrane organization controls receptor compartmentalization, protein interactions and intracellular trafficking, relatively small changes in sterol composition can have widespread consequences for cellular physiology.

Structural biology subsequently demonstrated that many sterol-binding proteins discriminate among closely related sterol structures with remarkable specificity (66, 67, 68, 69). Nuclear receptors, sterol-sensing domains, transport proteins and biosynthetic enzymes frequently recognize individual sterol conformations rather than a generic sterol scaffold (68, 70, 71). These observations indicate that structural information progressively introduced during biosynthesis is subsequently interpreted by multiple classes of sterol-binding proteins.

Structural variation also determines metabolic fate. Additional unsaturations or subtle changes in stereochemistry modify susceptibility to enzymatic and non-enzymatic oxidation, thereby influencing the spectrum of downstream metabolites that can subsequently be generated. The exceptional oxidative reactivity of 7-DHC provides one of the best-characterized examples of this principle (26, 72).

Collectively, these observations show that relatively subtle structural modifications acquired during cholesterol biosynthesis produce disproportionately large functional consequences. Membrane properties, molecular recognition and metabolic reactivity are therefore determined not simply by the presence of a sterol molecule, but by its precise chemical structure. This relationship between structural diversification and functional specialization provides the molecular basis for the regulatory roles of individual sterol intermediates discussed in the following sections.

### Desmosterol: A prototype regulatory sterol

Among post-lanosterol intermediates, desmosterol provides one of the clearest examples of how a biosynthetic sterol can acquire regulatory functions independent of cholesterol. As the immediate precursor of cholesterol in the Bloch pathway, it differs from cholesterol only by a double bond at carbon 24 of the side chain. For many years, this seemingly minor structural variation was considered biologically insignificant, and desmosterol was viewed simply as the final intermediate before cholesterol formation.

The first indication that this interpretation was incomplete came from human genetics. Desmosterolosis, caused by mutations in DHCR24, is characterized by marked desmosterol accumulation associated with severe developmental abnormalities (22). These observations demonstrated that desmosterol cannot fully substitute for cholesterol during embryonic development despite their close structural similarity, providing early evidence that biosynthetic sterols possess distinct physiological functions.

A major conceptual advance followed with the identification of desmosterol as an endogenous ligand of liver X receptors (LXRs) (10). This discovery established a direct link between cholesterol biosynthesis and transcriptional regulation by showing that a post-lanosterol intermediate can function as a signaling molecule rather than solely as a metabolic precursor. Subsequent studies identified desmosterol as one of the principal physiological activators of LXR signaling (73).

Its physiological importance became particularly evident in macrophages. Spann and colleagues demonstrated that desmosterol accumulates in cholesterol-loaded macrophages, where it simultaneously activates LXR-dependent cholesterol efflux and represses inflammatory gene expression (74). More recently, selective inhibition of DHCR24 increased endogenous desmosterol concentrations sufficiently to reproduce these anti-inflammatory effects and improve hepatic steatosis in experimental models (75, 76). These studies illustrate how redistribution of sterol flux can modify cellular phenotype through changes in a single biosynthetic intermediate.

Desmosterol also illustrates the remarkable selectivity of sterol recognition. Beyond its activity toward LXRs, it functions as an endogenous orthosteric agonist of RORγt, where it stabilizes receptor conformation and modulates the activity of synthetic allosteric ligands (77, 78). Thus, the introduction of a single double bond is sufficient to create selective interactions with distinct nuclear receptors.

More generally, desmosterol illustrates a principle that extends beyond this individual metabolite. The biological properties of biosynthetic sterols are determined by their chemical structure rather than by their proximity to cholesterol within the pathway. Because desmosterol abundance varies with tissue identity, differentiation state, metabolic stress, and pharmacological intervention (23), it functions both as a regulatory metabolite and as an indicator of sterol flux redistribution.

### Sterol composition shapes membrane signaling landscapes

The biological activities of sterol intermediates are not mediated exclusively through direct interactions with receptors or signaling proteins. An equally important mechanism arises from their ability to organize biological membranes. Because sterols are major structural components of eukaryotic membranes, changes in sterol composition modify the physicochemical environment in which membrane proteins, signaling complexes and intracellular trafficking pathways operate. Sterol signaling therefore emerges from the combined effects of molecular recognition and membrane organization.

This concept extends Konrad Bloch's original hypothesis that cholesterol biosynthesis progressively optimizes sterol structure for membrane function (4). Rather than behaving as interchangeable structural lipids, biosynthetic sterols differ in their capacity to condense membranes, stabilize lipid domains and regulate membrane dynamics. Consequently, membrane function reflects the composition of the sterol pool rather than cholesterol abundance alone.

Experimental studies support this view. Replacing cholesterol with biosynthetic sterol intermediates alters membrane packing, lipid order, permeability, curvature and the organization of cholesterol-rich membrane domains (24, 25, 64, 65, 79). These effects are highly sterol specific. Desmosterol preserves many membrane-condensing properties of cholesterol while remaining biophysically distinct, whereas zymosterol and 7-DHC produce more pronounced alterations in membrane organization (24, 25, 80). Different sterol compositions therefore generate distinct membrane environments.

The physiological consequences become particularly apparent in inherited disorders of cholesterol biosynthesis. In Smith–Lemli–Opitz syndrome, accumulation of 7-DHC disrupts membrane organization, alters lipid-domain architecture and impairs receptor function independently of cholesterol depletion (81, 82). These observations demonstrate that membrane dysfunction results primarily from replacement of cholesterol by structurally distinct sterols rather than from cholesterol deficiency itself.

Because membrane domains spatially organize signaling proteins, changes in sterol composition influence multiple signaling pathways simultaneously. The Hedgehog pathway provides one of the best-characterized examples. Activation of Smoothened (SMO) depends on membrane sterol composition and on the availability of several sterol species rather than cholesterol alone (83, 84, 85, 86, 87). Similar principles apply to other membrane-associated receptors, including EGFR, whose localization and signaling efficiency are influenced by membrane sterol organization (64, 88, 89).

Membrane remodeling therefore complements classical ligand-dependent signaling. Whereas individual sterols selectively regulate receptors such as LXRs or RORγt, the collective composition of the sterol pool determines the membrane landscape within which numerous signaling pathways operate. Redistribution of sterol flux thus influences cellular behaviour at both the molecular and supramolecular levels.

These observations illustrate how modest changes in sterol composition can produce disproportionately large biological effects. By simultaneously modifying membrane architecture and the spatial organization of signaling complexes, sterol flux rewiring reshapes the cellular environment in which signaling networks function. Changes in sterol composition therefore affect not only the activity of individual membrane proteins but also the spatial organization of signaling networks.

### Sterol intermediates participate in homeostatic feedback

The regulatory functions of biosynthetic sterols extend beyond membrane organization and receptor signaling. They also participate directly in the feedback mechanisms that control cholesterol biosynthesis itself. Rather than acting solely as metabolic intermediates, sterols contribute to the molecular systems that sense the state of the pathway and continuously adjust its activity.

A major advance in understanding this regulation came with the identification of the SREBP-SCAP-INSIG system, which coordinates the expression of genes involved in cholesterol synthesis and uptake (14, 90, 91). Under sterol-depleted conditions, SREBPs are transported from the endoplasmic reticulum (ER) to the Golgi apparatus for proteolytic activation. When sterol levels increase, sterol-dependent interactions between SCAP and INSIG retain the complex within the ER, thereby repressing transcription of cholesterogenic genes. Importantly, this regulatory machinery responds to the composition of the intracellular sterol pool rather than to cholesterol alone (92).

Feedback regulation also occurs at the level of enzyme stability. HMG-CoA reductase (HMGCR), the rate-limiting enzyme of the mevalonate pathway, undergoes INSIG-dependent ubiquitination and proteasomal degradation in response to sterol accumulation (93, 94, 95). Remarkably, lanosterol and several early biosynthetic intermediates promote HMGCR degradation more efficiently than cholesterol itself, demonstrating that biosynthetic sterols actively regulate flux through the pathway they help generate.

This regulatory system is itself dynamically controlled. INSIG-1 undergoes sterol-dependent ubiquitination and degradation (96), allowing continuous adjustment of pathway sensitivity according to changes in sterol composition. Cholesterol homeostasis therefore results from nested feedback loops that coordinate gene expression, enzyme turnover and sterol metabolism.

Human genetics and pharmacological studies provide complementary support for this model. Mutations affecting DHCR7, DHCR24, SC5D or EBP generate characteristic sterol profiles associated with distinct developmental disorders (17, 20, 21, 22, 97). Likewise, selective inhibition of distal cholesterol biosynthesis engages these feedback mechanisms differently according to the intermediates that accumulate (30, 38, 39). These observations indicate that homeostatic regulation monitors the organization of the sterol pool rather than the concentration of a single metabolite.

Together, these findings considerably broaden the classical view of cholesterol homeostasis. Biosynthetic sterols function simultaneously as metabolic intermediates and regulatory signals, allowing cholesterol biosynthesis to continuously adapt to changes in its own metabolic organization. This self-adjusting architecture provides a mechanistic basis for the remarkable plasticity of sterol metabolism discussed throughout this Review.

### Sterol composition defines the oxidative landscape

The consequences of sterol flux rewiring extend beyond membrane organization and receptor signaling. By altering the composition of the intracellular sterol pool, it also changes the repertoire of substrates available for oxidation. Cholesterol biosynthesis therefore influences not only membrane properties and signaling, but also the oxidative chemistry that can develop within a given cellular context.

Smith-Lemli-Opitz syndrome (SLOS) provides the clearest illustration of this principle. Loss of DHCR7 activity leads to the accumulation of 7-DHC, a sterol that undergoes free-radical oxidation several hundred-fold faster than cholesterol (20, 26, 72, 98). The resulting family of oxysterols contributes directly to oxidative stress, neuronal dysfunction and disease pathophysiology (97, 99, 100), demonstrating that changes in sterol composition can profoundly modify the biological consequences of oxidative metabolism.

More generally, oxidative metabolism reflects the interaction between two variables: the sterol substrates available for oxidation and the cellular redox environment. The same oxidative conditions can therefore generate different oxysterol repertoires from different sterol pools, whereas the same sterol substrate may give rise to distinct products depending on the cellular context (26, 63, 72, 98, 101). Sterol composition thus defines not only the current biochemical state of a cell but also its oxidative potential.

The 5,6-EC pathway provides another illustration of this principle. Formation of 5,6-ECs depends on both cholesterol availability and oxidative conditions, whereas their subsequent metabolism is strongly influenced by cellular antioxidant defenses. Tamoxifen promotes lipid peroxidation, cholesterol epoxidation and 5,6-EC accumulation, while the endogenous ChEH inhibitor DDA induces catalase expression and limits cholesterol epoxidation despite targeting the same EBP-DHCR7 complex (45, 46, 48). Thus, the biological outcome reflects the combined regulation of sterol metabolism and redox homeostasis rather than either process alone.

Genetic disorders, pharmacological interventions and physiological adaptations therefore modify oxidative metabolism by changing both the composition of the sterol pool and the conditions under which oxidation occurs (20, 30, 32, 33, 102, 103). Oxysterols and EChAs should consequently be viewed not as random products of lipid oxidation but as predictable outputs of specific sterol compositions exposed to defined redox environments.

Sterol signaling does not end with biosynthetic intermediates. Oxidative metabolism further diversifies these molecules, greatly expanding their structural and functional repertoire. This relationship establishes oxidative metabolism as a natural extension of sterol flux rewiring and provides the basis for the oxidative sterol networks discussed in the following sections.

## Oxidative diversification expands the sterol metabolic network

Generation of distinct sterol states does not mark the endpoint of cholesterol metabolism. Instead, it establishes the substrate landscape for a second level of metabolic organization in which biosynthetic sterols undergo enzymatic and non-enzymatic oxidation. These reactions greatly expand the molecular diversity generated during cholesterol biosynthesis, producing an interconnected network of signaling sterols (63, 101, 102, 104).

Enzymatic oxidation, mediated primarily by cytochrome P450 enzymes, generates regulatory oxysterols that participate in cholesterol homeostasis, immunity, development and cell differentiation through receptors including LXRs, RORs, EBI2 and SMO (63, 101, 104, 105, 106). In parallel, reactive oxygen species generate numerous non-enzymatic oxidation products whose formation depends on both sterol structure and the cellular redox environment.

Because individual sterols differ markedly in their chemical reactivity, each sterol state gives rise to a characteristic repertoire of oxidized metabolites. The extensive oxidation of 7-DHC in SLOS provides the best-characterized example of this principle, illustrating how alteration of a single biosynthetic step profoundly reshapes downstream oxysterol metabolism (20, 97, 98, 99, 100).

Importantly, most oxysterols are not metabolic endpoints. Many undergo further transformations, including hydroxylation, ketone formation, epoxide hydrolysis, sulfation, side-chain oxidation or conjugation, generating successive families of secondary, tertiary and higher-order metabolites (6, 102, 103). Cholesterol metabolism therefore develops through successive layers of chemical diversification rather than a single oxidative event.

This hierarchical organization considerably broadens the traditional view of oxysterol biology. Cholesterol biosynthesis determines the repertoire of sterol substrates, whereas oxidative metabolism transforms these substrates into interconnected families of signaling molecules. Oxidative sterol metabolism is therefore best viewed as a continuation of sterol flux rewiring rather than as a separate metabolic process.

Among these oxidative networks, the 5,6-EC pathway represents one of the most elaborate examples. Rather than generating a single bioactive metabolite, 5,6-ECs occupy a metabolic branch point from which successive enzymatic reactions produce an expanding family of EChAs with distinct, and often opposing, biological activities (6, 45, 102, 103). This pathway illustrates particularly well how oxidative metabolism extends the functional diversity generated during cholesterol biosynthesis and serves as the focus of the following section.

The epoxycholestanoid pathway illustrates how oxidative diversification extends beyond the formation of individual metabolites to generate an organized metabolic network in which successive products exert coordinated biological functions.

## The cholesterol-5,6-epoxide network as a metabolic Decision Hub

### 5,6-ECs: from oxidation products to metabolic branch points

5,6-ECs were originally regarded as oxidation products before progressively emerging as precursors of a distinct metabolic network. Rather than acting solely as signaling oxysterols, they serve as metabolic intermediates from which sterol metabolism diverges toward several downstream pathways generating metabolites with distinct, and often opposing, biological activities. The 5,6-EC pathway therefore illustrates how cholesterol oxidation can create new metabolic opportunities rather than simply modify cholesterol (6, 101, 102).

5,6-ECs are generated by oxidation of the Δ5 double bond of cholesterol through both free-radical and enzymatic mechanisms, yielding the two stereoisomers 5,6α-EC and 5,6β-EC (6, 9, 107). As discussed in the preceding section, their abundance reflects the combined influence of sterol metabolism and the cellular redox environment.

For many years, these epoxides were regarded primarily as cytotoxic products of lipid peroxidation (108). This view changed with the identification of ChEH and the demonstration that both 5,6-EC stereoisomers undergo extensive downstream metabolism (45). It became clear that the biological consequences of cholesterol epoxidation depend not simply on epoxide formation but on the metabolic pathways that subsequently utilize these intermediates.

These pathways generate metabolites with markedly different biological activities. 5,6α-EC modulates LXR transcriptional activity in a cell-dependent manner (109) and serves as the precursor of DDA, an endogenous steroidal alkaloid that promotes tumor cell differentiation, lethal autophagy and antitumor immunity (47, 48, 110, 111, 112, 113). Both stereoisomers can induce oxidative stress and cytotoxicity in a context-dependent manner, although their metabolic fates and biological effects are not identical (5, 6, 46, 47, 108, 114, 115).

Accordingly, the importance of 5,6-ECs lies less in their accumulation than in the metabolic choices they enable. By partitioning sterol flux toward pathways involved in differentiation, immune regulation, oxidative adaptation or tumor promotion, they function as a metabolic branch point where sterol flux is partitioned into distinct biological programs. The organization of these competing pathways is examined in the following sections.

### Cholesterol epoxide hydrolase: the first enzymatic checkpoint of the epoxycholestanoid network

If 5,6-ECs constitute the metabolic branch point of the EChA pathway, ChEH represents its first enzymatic checkpoint. By catalyzing the hydrolysis of 5,6-ECs into CT, ChEH determines whether sterol flux proceeds toward CT-derived metabolites or remains available for competing pathways, including DDA biosynthesis. Its biological importance therefore extends well beyond epoxide hydrolysis to the regulation of metabolic flux throughout the EChA network.

The discovery of ChEH emerged unexpectedly from pharmacology rather than classical sterol biochemistry. During the late 1970s and 1980s, several laboratories identified a high-affinity microsomal binding site for tamoxifen and related triphenylethylenes that was clearly distinct from the estrogen receptor (116, 117). This pharmacological entity, later termed the AEBS, recognized a remarkably broad spectrum of structurally diverse ligands (35, 118, 119), yet its physiological function remained unknown for more than 2 decades.

Identification of EBP as a component of the AEBS, followed by the demonstration that high-affinity ligand binding requires its association with DHCR7, established the first molecular connection between the AEBS and post-lanosterol sterol metabolism (30, 40, 41, 42). The decisive advance came when the EBP-DHCR7 complex was shown to catalyze hydrolysis of 5,6-ECs into CT, thereby identifying the AEBS as ChEH (45). This discovery established a direct biochemical link between cholesterol biosynthesis and oxidative sterol metabolism.

ChEH does more than generate CT. By controlling the consumption of 5,6-ECs, it regulates substrate availability for competing downstream pathways and thereby influences the balance between metabolites with distinct biological activities. CT itself undergoes further metabolism, notably into OCDO, creating a second branching point within the EChA pathway (6, 120). Thus, the biological consequences of cholesterol epoxidation depend not only on epoxide formation but also on how efficiently ChEH directs sterol flux through the network.

Pharmacological studies reinforce this concept. AEBS ligands simultaneously remodel post-lanosterol sterol biosynthesis, inhibit ChEH, alter intracellular 5,6-EC concentrations and redistribute flux through competing EChA pathways (46, 48, 121, 122). Their biological effects therefore arise from coordinated remodeling of interconnected metabolic pathways rather than inhibition of a single enzymatic activity.

This organization also has important translational implications. Compounds targeting EBP, DHCR7 or DHCR24 are increasingly being investigated in cancer, neurological disorders and metabolic disease (75, 76, 123, 124, 125, 126, 127, 128). Their effects on ChEH activity and downstream EChA metabolism have not been considered to dateñ. Because these proteins belong to the same metabolic network, pharmacological evaluation should extend beyond conventional sterol profiling to include 5,6-ECs, CT and downstream EChAs.

ChEH therefore occupies a strategic position within sterol metabolism. Positioned at the interface between cholesterol biosynthesis and oxidative metabolism, it governs the first metabolic partitioning event of the EChA pathway and thereby determines which downstream biological programs remain accessible to the cell. The consequences of this metabolic partitioning are examined in the following section.

### Metabolic partitioning within the 5,6-EC network

**Figure 3:**
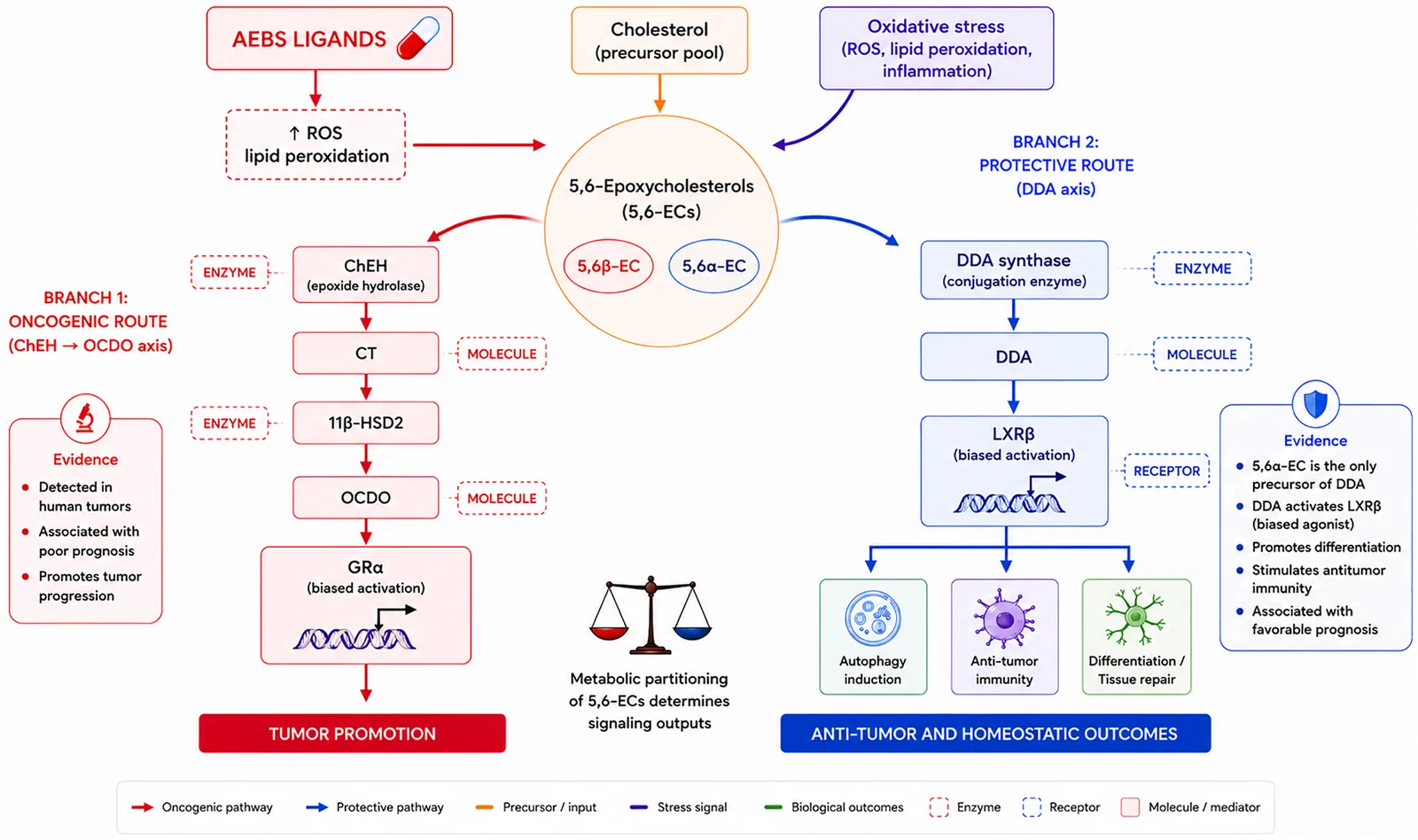
**Metabolic partitioning within the epoxycholestanoid (EChA) network determines cell fate.** Cholesterol 5,6-epoxides (5,6-ECs) constitute the primary branch point of the epoxycholestanoid (EChA) network, linking cholesterol metabolism, oxidative stress and cell-fate regulation. Cholesterol epoxidation is promoted by reactive oxygen species and lipid peroxidation, whereas AEBS ligands simultaneously perturb distal cholesterol biosynthesis, inhibit cholesterol epoxide hydrolase (ChEH), and increase cholesterol epoxidation. ChEH, formed by the EBP catalytic subunit associated with DHCR7, hydrolyzes 5,6-ECs into cholestane-3β,5α,6β-triol (CT), which can subsequently be oxidized by HSD11B2 to generate 6-oxo-cholestan-3β,5α-diol (OCDO, oncosterone), a biased GRα agonist associated with tumor progression. Alternatively, 5,6α-EC undergoes enzyme-catalyzed conjugation with histamine to generate dendrogenin A (DDA), an endogenous steroidal alkaloid that acts as a biased LXRβ agonist and promotes differentiation, lethal autophagy, lysosomal reprogramming, and antitumor immunity. The biological consequences of cholesterol epoxidation therefore depend less on the formation of 5,6-ECs than on their metabolic partitioning between competing EChA pathways.

The biological consequences of cholesterol 5,6-epoxidation depend less on the formation of 5,6-ECs than on the metabolic pathways that subsequently consume them. Once generated, 5,6-ECs enter a dynamic network in which competing enzymatic reactions partition sterol flux toward distinct EChAs with markedly different biological activities (Fig. 3). Thus, the functional output of the pathway reflects metabolic partitioning rather than the intrinsic properties of the epoxides themselves.

Under physiological conditions, ChEH catalyzes the hydrolysis of both 5,6α-EC and 5,6β-EC to generate CT, which represents a major metabolic fate of 5,6-ECs (8, 45). CT is not a metabolic endpoint but the precursor of additional EChAs, including OCDO following oxidation by 11β-HSD2/*HSD11B2* (6, 120). OCDO functions as a GRα-biased agonist that promotes breast cancer cell proliferation, tumor growth, and metastatic dissemination (120, 129).

An alternative pathway competes directly for the same substrate. Instead of hydrolysis, 5,6α-EC undergoes enzymatic conjugation with histamine to produce DDA, the first endogenous steroidal alkaloid identified in mammals (47). DDA promotes tumor cell differentiation, induces lethal autophagy, activates a selective LXRβ-dependent transcriptional program, reinforces antioxidant defenses through catalase induction and enhances anti-tumor immunity by stimulating the production of immunogenic extracellular vesicles (5, 48, 111, 112, 130).

These examples illustrate a central feature of the EChA network: biological functions emerge from the distribution of sterol flux between competing metabolic pathways rather than from the activity of a single metabolite. Depending on enzyme expression, substrate availability, redox status, differentiation state and tissue context, identical amounts of 5,6-ECs can generate profoundly different EChA repertoires. Because substrate availability is itself determined by upstream sterol flux rewiring, changes in cholesterol biosynthesis propagate directly into downstream EChA metabolism.

Cancer provides a striking example of this metabolic redistribution. Compared with normal tissues, malignant cells display a profound remodeling of the EChA network characterized by loss of DDA biosynthesis together with maintenance, or increased flux, through CT-dependent pathways leading to OCDO production (5, 47, 120, 131). This shift favors proliferation, tumor progression and immune escape at the expense of differentiation and tissue homeostasis.

More broadly, the EChA pathway differs from most classical oxysterol pathways. Whereas many oxysterols exert their biological activities directly, 5,6-ECs initiate successive enzymatic transformations that generate secondary, tertiary and quaternary metabolites with increasingly specialized functions (6, 103). In this respect, the EChA pathway resembles endogenous lipid mediator systems such as the eicosanoid network, in which a common precursor is partitioned among competing enzymatic pathways to generate mediators with complementary or opposing biological activities (132, 133, 134, 135).

Accordingly, 5,6-ECs should be viewed not simply as oxidized cholesterol derivatives but as the origin of an organized sterol signaling network. Their importance lies in their capacity to partition sterol flux among competing metabolic pathways, thereby linking cholesterol metabolism to cell fate through dynamic metabolic competition.

### The hierarchical organization of the epoxycholestanoid network

The EChA pathway reveals an organizational principle that extends beyond the metabolism of 5,6-ECs themselves. Rather than generating isolated signaling molecules, successive enzymatic reactions progressively diversify cholesterol-derived metabolites into a hierarchically organized network. This framework classifies EChAs according to their biosynthetic relationships and illustrates how new biological functions emerge through sequential metabolic transformations rather than through the action of individual metabolites alone (6, 102).

Within this hierarchy, 5,6-ECs constitute the primary EChAs because they arise directly from cholesterol oxidation. Their hydrolysis by ChEH generates the secondary EChA CT, whereas conjugation of 5,6α-EC with histamine produces the secondary EChA DDA (45, 47). CT can undergo oxidation by HSD11B2 to generate the tertiary EChA OCDO, or side-chain hydroxylation by CYP27A1 to produce 27H-CT, another tertiary EChA. Subsequent CYP27A1-dependent hydroxylation of OCDO generates the quaternary EChA 27H-OCDO (6, 120, 136), illustrating how successive reactions continue to expand the structural diversity of the pathway (6).

This hierarchical organization is biologically significant because every metabolic transformation modifies the information carried by the molecule. Epoxide hydrolysis, histamine conjugation, ketone formation or side-chain hydroxylation alter receptor recognition, membrane interactions, metabolic stability and susceptibility to further biotransformation. Consequently, biological functions emerge progressively as sterol metabolites move through the network rather than being determined solely by the structure of the initial oxidation product.

Unlike the traditional view of oxysterol biology, in which individual metabolites are generally considered independently, the EChA pathway emphasizes the importance of metabolic relationships. The activity of a given EChA depends not only on its intrinsic properties but also on its biosynthetic origin, its downstream metabolites and the partitioning of sterol flux throughout the network.

More broadly, the EChA pathway suggests that oxidative sterol metabolism may follow organizational principles shared with other endogenous lipid mediator systems while introducing an additional level of complexity. Unlike eicosanoids, which arise from the partitioning of a common precursor, the EChA network is itself embedded within the broader organization of cholesterol biosynthesis. Metabolic partitioning therefore operates at two hierarchical levels: first during the establishment of sterol states, and subsequently within the EChA network itself. This nested organization considerably expands the capacity of sterol metabolism to generate biological diversity.

Whether similar hierarchically organized networks exist for other sterol oxidation pathways remains unknown. However, continuing advances in sterolomics, isotope tracing and metabolic flux analysis strongly suggest that additional sterol families organized according to comparable principles are likely to emerge.

**Figure 4:**
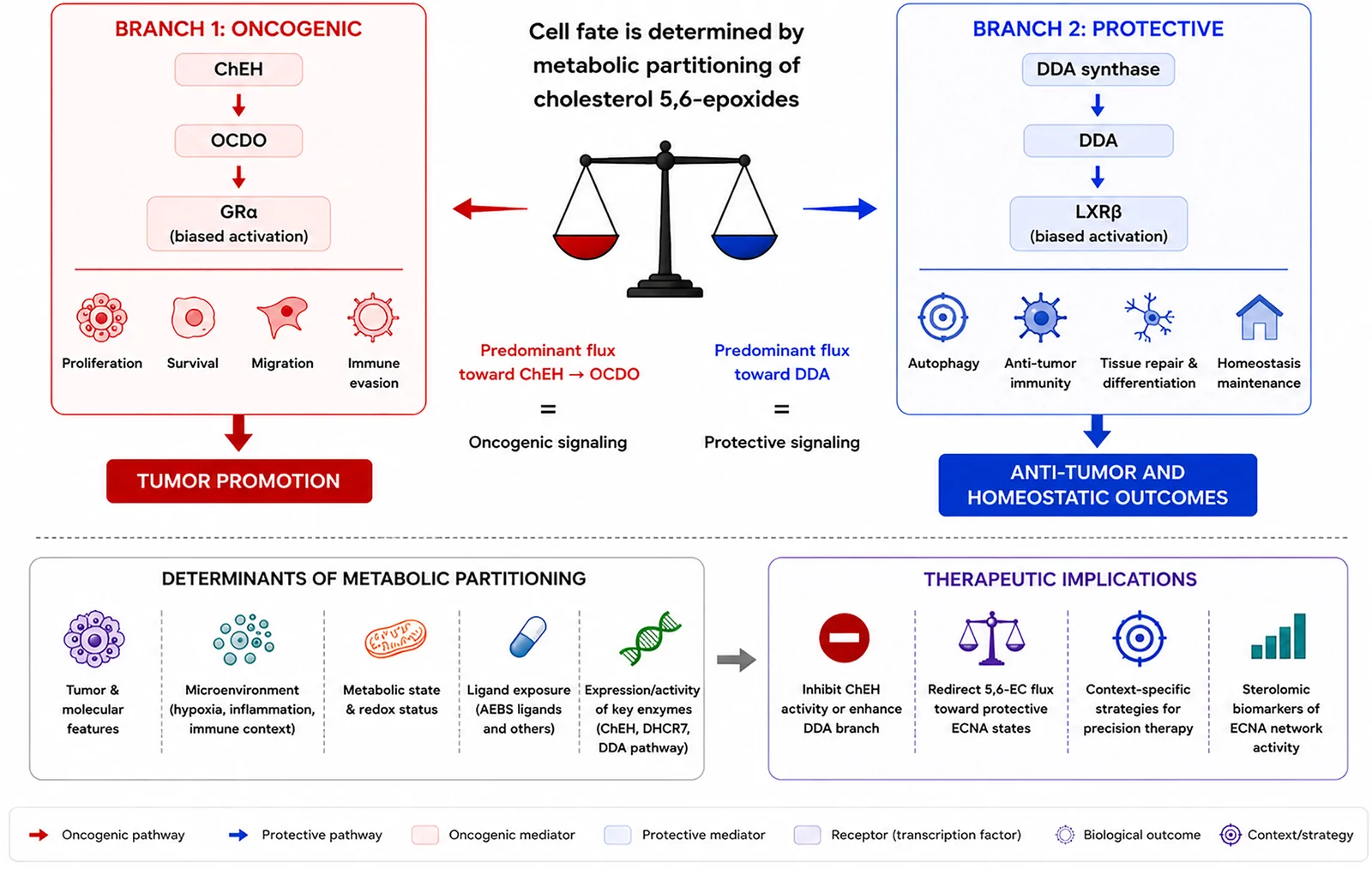
**Metabolic partitioning within the epoxycholestanoid (EChA) network determines divergent biological outcomes.** The biological consequences of cholesterol 5,6-epoxidation depend on how metabolic flux is partitioned between competing branches of the epoxycholestanoid (EChA) network. Increased flux through the cholesterol epoxide hydrolase (ChEH)-dependent pathway promotes conversion of cholesterol 5,6-epoxides into cholestane-3β,5α,6β-triol (CT) and subsequently 6-oxo-cholestan-3β,5α-diol (OCDO), leading to biased activation of glucocorticoid receptor α (GRα) signaling associated with tumor progression. Conversely, metabolic routing of 5,6α-epoxycholesterol toward dendrogenin A (DDA) biosynthesis promotes biased activation of liver X receptor β (LXRβ), inducing differentiation, lethal autophagy, tissue repair and antitumor immunity. The balance between these competing pathways is influenced by multiple factors, including tumor genotype, tissue microenvironment, oxidative status, pharmacological agents, and expression of key metabolic enzymes. This model illustrates how sterol flux rewiring regulates cell fate through metabolic partitioning rather than through the activity of a single metabolite and highlights potential therapeutic strategies aimed at restoring protective EChA signaling.

Collectively, these pathways illustrate how sterol flux rewiring generates interconnected signaling networks whose physiological and pathological consequences are summarized in Figure 4 and discussed in the following section.

## Sterol flux rewiring across physiology and disease

The preceding sections show that sterol metabolism is better understood as a flexible metabolic network than as a linear pathway dedicated to cholesterol production. Redistribution of sterol flux reshapes not only the abundance of biosynthetic intermediates but also the production of oxysterols, EChAs and other downstream metabolites, thereby modifying the biochemical environment in which cells respond to developmental, physiological and pathological cues.

Some of the strongest evidence for this view comes from stable-isotope tracing studies demonstrating that post-lanosterol cholesterol biosynthesis does not proceed through two invariant Bloch and Kandutsch–Russell pathways. Instead, individual tissues combine alternative biosynthetic routes according to their physiological requirements, generating reproducible tissue-specific sterol profiles that remain largely preserved in cultured cells (61). Sterol pathway organization therefore emerges as a regulated property of cellular physiology rather than a fixed feature of cholesterol biosynthesis.

This organization has important biological consequences. Because sterol intermediates differ in their biophysical properties, signaling activities and metabolic fates, even modest changes in flux distribution can remodel membrane function, oxidative metabolism and downstream signaling without necessarily producing major changes in cholesterol abundance.

The examples discussed below illustrate how this principle applies across diverse biological settings. Although cancer, immunity, neurobiology and ageing differ profoundly in their underlying mechanisms, each is associated with characteristic patterns of sterol flux redistribution that reshape the cellular sterol architecture and contribute to biological function or disease.

### Cancer reveals the biological consequences of sterol flux rewiring

Cancer provides one of the clearest examples of sterol flux rewiring. Although activation of the mevalonate pathway is a common feature of many tumors, malignant cells also remodel post-lanosterol sterol metabolism, generating sterol states that support proliferation, metabolic adaptation, and immune escape through mechanisms that cannot be explained by cholesterol abundance alone (16, 131).

Some of the earliest experimental evidence came from studies by Lasunción and colleagues, who demonstrated that pharmacological inhibition at different positions within distal cholesterol biosynthesis produced distinct sterol profiles associated with markedly different effects on cell cycle progression and proliferation in human cancer cells (27, 28, 29). These pioneering studies established that biosynthetic sterol intermediates are not simply passive markers of pathway inhibition but active determinants of tumor cell phenotype.

Hedgehog signaling provides another compelling illustration of this principle. Smoothened (SMO), the central signal transducer of the Hedgehog pathway, is now recognized as a sterol-regulated receptor rather than a simple downstream effector of Patched. Cholesterol directly activates SMO (84), while several endogenous oxysterols, including 20(S)-hydroxycholesterol and 24(S),25-epoxycholesterol, modulate its activity through distinct sterol-binding sites (83, 87, 137, 138, 139, 140). Conversely, B-ring oxysterols generated during the oxidation of 7-DHC can inhibit SMO through mechanisms distinct from those of both cyclopamine and activating side-chain oxysterols (85, 140). Collectively, these studies demonstrate that Hedgehog signaling is determined not by cholesterol alone but by the composition and oxidative fate of the intracellular sterol pool.

Recent work further illustrates how oxidative diversification amplifies the biological consequences of sterol flux rewiring. In Smith-Lemli-Opitz syndrome and other conditions associated with 7-DHC accumulation, free-radical oxidation generates a complex family of bioactive oxysterols, including 3β,5α-dihydroxycholest-7-en-6-one (DHCEO), which contribute to abnormal neuronal differentiation and developmental defects (72, 141, 142). Although these mechanisms were initially characterized in developmental disorders, they establish a broader principle directly relevant to cancer: alterations in post-lanosterol sterol metabolism reshape signaling not only through the accumulation of biosynthetic intermediates but also through the secondary metabolites generated by their oxidative diversification.

The therapeutic implications of this sterol dependence are illustrated in Hedgehog-driven tumors. In medulloblastoma models, pharmacological inhibition of cholesterol biosynthesis using statins suppresses Hedgehog signaling, inhibits tumor growth and potentiates the efficacy of the SMO antagonist vismodegib (143, 144). These findings demonstrate that pharmacological remodeling of sterol metabolism can influence oncogenic signaling by altering the sterol environment required for pathway activation.

Additional examples have revealed therapeutically exploitable metabolic vulnerabilities within distal cholesterol biosynthesis. APC-mutant colorectal cancers display a marked dependence on EBP that is selectively targeted by Truncated APC-Selective Inhibitors (TASINs), which disrupt post-lanosterol sterol metabolism and selectively eliminate APC-deficient cells while sparing normal tissues (123, 124, 145). Rather than resulting from generalized cholesterol depletion, this selective toxicity reflects the generation of a tumor-specific sterol state incompatible with cancer-cell survival.

An equally striking example has recently emerged from studies of ferroptosis. Although EBP and DHCR7 catalyze consecutive reactions within distal cholesterol biosynthesis, inhibition of these neighboring enzymes produces opposite biological outcomes. EBP inhibition decreases endogenous 7-DHC, sensitizes tumor cells to ferroptosis and suppresses tumor growth, whereas DHCR7 inhibition promotes 7-DHC accumulation, protects tumor cells from ferroptosis and enhances metastatic dissemination (127, 128). These contrasting phenotypes illustrate how subtle changes in sterol flux redirect metabolism toward sterol states differing in their content of oxidation-sensitive and radical-trapping sterols.

The EChA pathway extends this concept beyond cholesterol biosynthesis itself. Malignant transformation is accompanied by a profound redistribution of epoxycholestanoid metabolism characterized by loss of DDA biosynthesis together with preservation, or even enhancement, of CT-dependent metabolism leading to OCDO production (6, 47, 120, 131). This metabolic partitioning shifts signaling away from differentiation, antioxidant protection and antitumor immunity while maintaining a GRα-dependent program that promotes proliferation, tumor progression and metastatic dissemination.

Taken together, these observations indicate that tumor cells depend on specific sterol states rather than on elevated cholesterol synthesis alone. Distal cholesterol biosynthetic enzymes should therefore be viewed not simply as isolated pharmacological targets but as regulatory nodes whose manipulation redistributes sterol flux across interconnected signaling networks. Future therapeutic strategies will likely benefit from integrating conventional measures of antitumor efficacy with comprehensive sterolomic analyses capable of simultaneously characterizing biosynthetic sterols, 7-DHC-derived oxysterols, 5,6-epoxycholesterols and downstream epoxycholestanoids.

### Sterol flux rewiring shapes immune and inflammatory responses

Immune activation is accompanied by extensive remodeling of sterol metabolism that extends well beyond changes in cholesterol abundance. Activation of innate and adaptive immune cells reorganizes cholesterol biosynthesis, intracellular sterol trafficking and oxidative sterol metabolism, coupling metabolic adaptation to inflammatory signaling, cellular differentiation and host defense (7, 74, 105, 146). More broadly, immune-cell activation requires coordinated metabolic reprogramming involving glycolysis, mitochondrial metabolism, amino-acid utilization and lipid metabolism (147, 148, 149, 150). Sterol flux rewiring can therefore be viewed as one component of this broader immunometabolic adaptation.

Macrophages provide one of the clearest examples of this metabolic plasticity. During Toll-like receptor (TLR) activation, selective suppression of CYP51A1 redirects post-lanosterol metabolism, leading to the accumulation of lanosterol and 24,25-dihydrolanosterol (151). These intermediates attenuate type I interferon signaling while enhancing phagocytosis and antibacterial activity, demonstrating that inflammatory activation establishes sterol states specifically adapted to innate immune function.

Desmosterol illustrates a complementary mechanism. In cholesterol-loaded macrophages, endogenous desmosterol accumulates sufficiently to activate LXR-dependent transcriptional programs while simultaneously repressing inflammatory gene expression (74). Selective inhibition of DHCR24 reproduces this anti-inflammatory sterol state by increasing endogenous desmosterol without substantially altering cholesterol concentrations, leading to reduced hepatic inflammation in experimental models (75, 76).

Oxidative sterol metabolism further expands this regulatory landscape. Oxysterols such as 25-HC, 7α,25-diHC and 24(S)-HC regulate innate and adaptive immunity through LXRs, EBI2, RORs and other sterol-responsive pathways (63, 102, 105, 106). Their production depends on the sterol state established upstream, linking biosynthetic remodeling to immune signaling through successive metabolic transformations.

The EChA pathway extends this integration to antitumor immunity. Redistribution of 5,6-EC metabolism modifies the balance between metabolites that favor tumor progression and those that stimulate immune surveillance. DDA promotes tumor-cell differentiation while inducing the production of BMP-enriched extracellular vesicles that enhance dendritic-cell maturation, antigen presentation, CD4^+^ and CD8^+^ T-cell activation, and the efficacy of immune checkpoint blockade (110, 111). Thus, rewiring a single oxidative branch of sterol metabolism simultaneously remodels tumor-cell behavior and both innate and adaptive immune responses.

Collectively, these studies show that immune function depends on the organization of sterol metabolism rather than on cholesterol availability alone. Biosynthetic sterols, oxysterols and EChAs act as interconnected regulatory systems whose coordinated remodeling shapes inflammatory signaling, immune-cell differentiation and host defense.

### Sterol flux rewiring in the nervous system: from developmental disease to regeneration

The central nervous system provides one of the clearest examples of the importance of sterol metabolism beyond cholesterol production. Because circulating cholesterol crosses the blood-brain barrier only minimally, virtually all brain cholesterol is synthesized locally, making neural tissues particularly dependent on the precise regulation of post-lanosterol sterol metabolism (152). Consequently, relatively small changes in sterol composition can profoundly influence neurodevelopment, myelination, synaptic function and neuronal survival.

This vulnerability first became apparent through inherited disorders of cholesterol biosynthesis. Smith-Lemli-Opitz syndrome, caused by mutations in DHCR7, was initially interpreted as a consequence of cholesterol deficiency. This view changed after the recognition that the accumulated precursor 7-DHC is exceptionally prone to free-radical oxidation, generating a complex mixture of bioactive oxysterols that contribute to oxidative stress, mitochondrial dysfunction, impaired neuronal differentiation and developmental abnormalities (20, 72, 98, 99, 100, 153). SLOS therefore illustrates how alterations in sterol composition reshape the neuronal biochemical environment, with consequences extending well beyond reduced cholesterol availability.

A similar concept is emerging in more common neurological disorders. Altered cholesterol biosynthesis, intracellular sterol trafficking and oxidative sterol metabolism have all been implicated in Alzheimer's disease, Parkinson's disease, Huntington's disease and multiple sclerosis (7, 70, 102). Although the molecular mechanisms differ among these disorders, they collectively suggest that neuronal homeostasis depends on maintaining an appropriate balance among sterol metabolites rather than on cholesterol alone.

Perhaps the most compelling evidence that sterol metabolism can be therapeutically redirected comes from studies of remyelination. Partial inhibition of EBP promotes the accumulation of 8,9-unsaturated sterol intermediates that stimulate oligodendrocyte precursor differentiation and enhance myelin repair (125). Remarkably, chemically unrelated inhibitors of CYP51, TM7SF2 and EBP converge toward the same promyelinating sterol profile despite targeting different enzymes (126). Similar effects have subsequently been reported for teriflunomide (154), while recent medicinal chemistry efforts have produced brain-penetrant EBP inhibitors specifically optimized to promote remyelination (155). These studies indicate that regenerative efficacy depends less on inhibition of a particular enzyme than on the sterol state created by metabolic reorganization.

Taken together, developmental disorders and regenerative therapies reveal two complementary aspects of sterol flux rewiring. Disturbance of sterol homeostasis can generate pathological sterol states that compromise neuronal function, whereas controlled remodeling of the same metabolic network can create regenerative sterol states that promote repair. The nervous system therefore illustrates particularly well how manipulating sterol composition, rather than simply restoring cholesterol levels, may offer new therapeutic opportunities.

### Aging reveals the long-term dynamics of sterol flux rewiring

Ageing provides a unique perspective on sterol metabolism because, unlike genetic disorders or pharmacological interventions, it reflects the cumulative effects of metabolic remodeling over decades. Throughout adult life, cholesterol biosynthesis, oxidative sterol metabolism and sterol signaling are continuously adjusted in response to changing physiological demands. Sterol flux rewiring therefore appears not only as a response to disease but also as an intrinsic component of long-term biological adaptation.

This gradual remodeling cannot be explained simply by changes in cholesterol abundance. Ageing is accompanied by progressive alterations in biosynthetic sterol profiles, increased oxidative diversification and changes in membrane composition that influence mitochondrial function, inflammatory responses, autophagy and cellular stress resistance (7, 9, 102, 156). Rather than reflecting a uniform decline in cholesterol metabolism, these changes suggest a continuous evolution of sterol states throughout life.

Oxidative sterol metabolism exemplifies this progressive transition. Age-associated accumulation of oxysterols such as 7-ketocholesterol and 7β-hydroxycholesterol has been associated with chronic inflammation, oxidative stress and mitochondrial dysfunction, whereas other sterol metabolites, including desmosterol, 24(S)-HC and 25-HC, participate in adaptive pathways regulating lipid metabolism, immune homeostasis and cellular resilience through receptors such as LXRs, EBI2, and RORs (9, 10, 74, 105, 157, 158, 159). Ageing therefore reflects not simply an increase in sterol oxidation, but a progressive reorganization of sterol signaling networks.

This perspective raises an important possibility. Functional decline may result not only from the accumulation of molecular damage but also from a gradual loss of metabolic flexibility. If sterol flux rewiring contributes to tissue adaptation throughout life, ageing could in part reflect a reduced capacity to generate sterol states that support repair, stress resistance and homeostasis. Recent remyelination studies, in which distinct pharmacological interventions converge toward regenerative sterol profiles, suggest that at least some of this metabolic plasticity can be restored experimentally (125, 126, 154, 155).

Viewed from this perspective, ageing is not simply associated with progressive deterioration of cholesterol homeostasis. Rather, it can be considered a lifelong remodeling of interconnected sterol networks whose adaptive capacity gradually becomes constrained. Determining how sterol states evolve during healthy and pathological ageing, and whether beneficial sterol configurations can be restored, now represents an important challenge for sterol biology.

## Sterol flux rewiring: Toward a new paradigm in sterol biology

### From cholesterol biosynthesis to sterol network biology

For more than two centuries, cholesterol metabolism has provided one of the clearest paradigms in biochemistry. From Chevreul's identification of *cholestérine* in 1816 to the pioneering contributions of Konrad Bloch, Brown and Goldstein, successive discoveries established cholesterol biosynthesis as a highly regulated metabolic pathway responsible for producing an essential membrane lipid while maintaining cellular homeostasis (1, 4, 14). This framework has profoundly shaped our understanding of lipid metabolism and remains one of the major achievements of modern biochemistry.

Collectively, however, the evidence reviewed here indicates that cholesterol represents only one outcome of a much broader metabolic organization. Throughout cholesterol biosynthesis, structurally related sterols acquire distinct biophysical properties, metabolic reactivities and signaling capacities that influence membrane organization, receptor activation, intracellular trafficking and oxidative metabolism. Cholesterol biosynthesis therefore appears less as a pathway ending with cholesterol than as a dynamic process that continuously generates a changing sterol organization.

Within this framework, metabolic flux emerges as a central regulatory variable. Genetic variation, developmental programs, oxidative stress and pharmacological intervention do not simply alter the rate of cholesterol synthesis. They redistribute metabolic intermediates, modify access to downstream oxidative pathways and reshape the repertoire of signaling sterols available to the cell. Cellular physiology is consequently influenced not only by the presence of individual metabolites but also by the organization of the metabolic network that connects them.

This perspective helps reconcile observations that have often been considered independently. Developmental disorders caused by defects in cholesterol biosynthesis, tissue-specific sterol signatures revealed by isotope-tracing studies, the identification of endogenous ligands for LXRs, RORs and Smoothened, the emergence of oxysterols and epoxycholestanoids as signaling molecules, and the growing number of therapeutic strategies targeting distal cholesterol biosynthesis can all be interpreted as manifestations of a common organizational principle. Rather than acting as isolated metabolites, sterols function within an interconnected metabolic system whose composition changes according to physiological context.

In many respects, this interpretation extends Konrad Bloch's evolutionary hypothesis. Bloch proposed that cholesterol biosynthesis progressively optimized sterol structure to produce an ideal membrane molecule (4). Current evidence instead suggests that evolution may also have optimized the organization of the pathway itself. Successive biosynthetic and oxidative transformations progressively increase molecular diversity, thereby creating multiple opportunities for regulation, signaling and adaptation long after cholesterol has been synthesized.

Importantly, this conceptual evolution has not arisen solely from the discovery of new sterol metabolites. It has also been driven by remarkable advances in analytical chemistry, mass spectrometry, structural biology and metabolic flux analysis, which now allow sterol metabolism to be investigated at an unprecedented level of resolution. These technologies increasingly reveal cholesterol metabolism as a distributed and adaptive network whose organization would have remained invisible using conventional biochemical approaches.

The concept of sterol flux rewiring therefore offers more than an alternative description of cholesterol biosynthesis. It provides a framework in which biosynthetic sterols, oxysterols and epoxycholestanoids are viewed as interconnected components of an adaptive signaling network linked through the continuous redistribution of metabolic flux. Similar principles govern other endogenous lipid mediator systems, including eicosanoids, sphingolipids and endocannabinoids, suggesting that network organization may represent a general property of lipid signaling rather than an exception specific to sterols.

Ultimately, the challenge is no longer simply to understand how cells synthesize cholesterol, but how they continuously reorganize their sterol architecture in response to developmental, physiological and pathological demands. Addressing this question will require experimental approaches capable of reconstructing sterol metabolism as an integrated and dynamic network rather than as a succession of isolated reactions. The methodological implications of this transition are considered in the following section.

### Toward integrated sterol-state analysis

If sterol metabolism is organized as a dynamic network rather than as a linear biosynthetic pathway, the experimental questions also change. Instead of quantifying cholesterol or a limited number of selected metabolites, future studies will increasingly need to characterize the composition, dynamics and organization of entire sterol networks. Cholesterol measurements will undoubtedly remain clinically valuable, but they capture only part of the metabolic information generated by sterol flux rewiring.

This transition is already becoming technically feasible. Advances in mass spectrometry have considerably expanded the scope of sterolomics, allowing the simultaneous quantification of biosynthetic sterols, oxysterols, steroidal alkaloids, sulfated sterols, bile-acid precursors and numerous additional sterol metabolites within a single biological sample (6, 160, 161, 162, 163, 164). Rather than providing isolated metabolite measurements, these approaches reveal coordinated remodeling of sterol metabolism that remains largely invisible when cholesterol is considered alone.

Beyond metabolite profiling, stable-isotope tracing has demonstrated that sterol metabolism is intrinsically dynamic. Mitsche and colleagues showed that individual tissues maintain characteristic sterol compositions by combining Bloch and Kandutsch-Russell reactions in different proportions rather than following invariant biosynthetic routes (61). These studies emphasize that metabolic flux, together with metabolite abundance, contributes to defining tissue-specific sterol states.

Recent developments are extending this resolution even further. Imaging mass spectrometry and spatial sterolomics now allow sterol metabolism to be visualized directly within intact tissues, revealing regional metabolic heterogeneity that cannot be resolved by conventional biochemical analyses (165). Single-cell lipidomics, multimodal imaging and spatial metabolomics are beginning to provide comparable information at the cellular level (166, 167). Combined with structural biology, isotope tracing, computational modelling and systems biology, these approaches should eventually allow reconstruction of sterol organization across cells, tissues and whole organisms.

A similar conceptual transition has already transformed several other areas of biology. Transcriptomics shifted attention from individual genes to coordinated gene-expression programs, proteomics revealed functional protein networks, and metabolomics redefined intermediary metabolism as an integrated system rather than a collection of isolated reactions. Sterol biology now appears ready to undergo a comparable evolution. The objective is no longer simply to quantify cholesterol, desmosterol or individual oxysterols, but to define characteristic sterol-state signatures associated with specific physiological, developmental and pathological conditions.

Achieving this goal will require more than measuring larger numbers of metabolites. Sterol composition will need to be interpreted together with metabolic flux, enzyme activity, redox status, tissue architecture and cellular context to reconstruct the dynamic organization of sterol metabolism. In this sense, sterol states should be viewed not merely as analytical profiles but as integrated functional phenotypes.

Ultimately, sterol-state signatures may become biologically meaningful descriptors of cellular identity, metabolic adaptation, disease progression and therapeutic response, particularly when integrated with transcriptomic, proteomic and spatial multi-omic data. In this perspective, sterolomics becomes more than an analytical technology. It provides the experimental foundation for a future precision sterol medicine in which diagnosis, patient stratification and therapeutic intervention are guided by the organization and dynamics of sterol metabolism rather than by cholesterol concentration alone.

### From enzyme inhibition to sterol network reprogramming

Viewing sterol metabolism as an adaptive metabolic network rather than a linear biosynthetic pathway profoundly changes the rationale for therapeutic intervention. Traditionally, drugs targeting cholesterol metabolism have been developed to inhibit individual enzymes or reduce cholesterol synthesis. The framework developed throughout this Review suggests a different objective. Therapeutic benefit may arise not simply from suppressing cholesterol production, but from redirecting sterol flux toward metabolic configurations that restore physiological functions or prevent pathological adaptation.

Several recent studies already support this concept. In APC-mutant colorectal cancer, TASIN compounds exploit a selective dependence on EBP to generate a sterol environment incompatible with tumor-cell survival rather than simply lowering cholesterol (123, 124, 168). Likewise, inhibition of EBP or DHCR7 produces opposite effects on ferroptosis and tumor progression because each perturbation generates a different post-lanosterol sterol composition (127, 128). These observations illustrate that biological outcome depends primarily on the sterol state established after metabolic rewiring rather than on the identity of the inhibited enzyme itself.

The same principle is emerging in regenerative neurology. Partial inhibition of EBP, CYP51 or TM7SF2 promotes oligodendrocyte differentiation and remyelination despite targeting different enzymes within cholesterol biosynthesis (125, 126, 154, 155). The common regenerative phenotype reflects convergence toward a shared sterol state rather than inhibition of a specific enzymatic reaction, further supporting the view that therapeutic efficacy depends on network organization.

The EChA pathway provides another illustration of this principle. Modulating metabolic partitioning at the level of ChEH or downstream EChA metabolism redirects sterol flux between differentiation-promoting and tumor-promoting signaling pathways without directly targeting a single receptor or signaling cascade (6, 48, 111). Here again, therapeutic intervention acts by reshaping the organization of a metabolic network rather than by modifying the concentration of an isolated metabolite.

These examples also have important implications for drug development. Distal cholesterol biosynthetic enzymes are embedded within highly interconnected metabolic systems linking sterol biosynthesis, intracellular trafficking, oxidative metabolism and epoxycholestanoid production. Pharmacological inhibition therefore rarely affects a single biochemical reaction. Instead, it redistributes metabolic flux throughout multiple branches of the sterol network. Evaluating these compounds solely through measurements of cholesterol synthesis or target inhibition is therefore unlikely to capture their full biological consequences. Comprehensive sterolomic analyses, combined whenever possible with metabolic-flux measurements, should become an integral component of preclinical and clinical evaluation of drugs targeting sterol metabolism.

More broadly, this perspective considerably expands the range of possible therapeutic strategies. Selective enzyme inhibitors, allosteric modulators, endogenous sterol metabolites, dietary interventions and redox-based approaches may ultimately be combined to stabilize beneficial sterol states or destabilize pathological ones. In this context, sterol metabolism becomes a programmable metabolic system whose organization, rather than simply its activity, can be manipulated for therapeutic benefit.

Ultimately, the transition from enzyme inhibition to sterol network reprogramming represents more than a change in pharmacological strategy. It reflects a broader shift in therapeutic philosophy. Rather than attempting to block individual metabolic reactions, future interventions may increasingly seek to reshape the organization of sterol metabolism itself so that the network adopts a physiological configuration incompatible with disease. If the concept of sterol flux rewiring proves broadly applicable, it may provide the conceptual basis for an entirely new class of metabolism-based therapies.

## Outstanding questions and future directions

The concept of sterol flux rewiring does not conclude the study of cholesterol metabolism; it redefines its most important questions. Rather than proposing a definitive model, it provides a framework for investigating sterol metabolism as a dynamic and adaptive system whose organization remains only partially understood. Progress will increasingly depend on integrating genetics, analytical chemistry, structural biology, cell biology, metabolism and computational approaches into a common experimental framework capable of capturing sterol metabolism in both space and time.

One of the most fundamental questions concerns the nature of sterol states themselves. Although characteristic sterol signatures accompany many physiological and pathological conditions, it remains unknown whether these states represent discrete metabolic configurations, continuously evolving distributions, or stable attractors generated by network dynamics. Determining how sterol states are established, maintained, and remodeled, and how they differ among tissues, developmental stages, and environmental conditions will require quantitative integration of biosynthetic sterols, oxysterols, epoxycholestanoids and metabolic flux within the same experimental framework.

A second challenge is to understand how cells interpret changes in sterol organization. Numerous proteins recognize individual sterols, including LXRs, RORs, EBI2, Smoothened and sterol-sensing domain-containing proteins (63, 83, 105, 158, 159). Yet many cellular responses, including membrane remodeling, receptor signaling and oxidative adaptation, appear to depend on the collective properties of the sterol pool rather than on individual metabolites alone. Whether cells primarily sense specific sterols or emergent properties of sterol organization remains an open question.

The remarkable diversity of sterol metabolism also remains incompletely explored. The discovery of signaling-active biosynthetic intermediates, regulatory oxysterols and the EChA family suggests that many additional sterol-derived metabolites remain to be identified (6, 7, 63). Comparable metabolic networks may also exist for phytosterols, fungal sterols and other sterol families, extending the concept of hierarchical sterol metabolism well beyond cholesterol itself.

These biological questions are closely linked to translational opportunities. Can characteristic sterol-state signatures become clinically useful biomarkers for diagnosis, prognosis or therapeutic monitoring? More importantly, can they evolve into network biomarkers that capture the functional organization of sterol metabolism rather than the concentration of individual metabolites? Addressing these questions will require integrated sterolomics combined with measurements of metabolic flux, redox state, tissue architecture and cellular context.

Answering such questions will depend on continued technological innovation. High-resolution sterolomics, isotope tracing, imaging mass spectrometry, single-cell lipidomics and computational systems biology are rapidly transforming our ability to characterize sterol metabolism. Rather than generating increasingly long lists of metabolites, these approaches should ultimately allow reconstruction of dynamic sterol networks across cells, tissues and organisms.

Perhaps the most significant implication of this work is conceptual. For decades, sterol biology has largely progressed by discovering new molecules and assigning individual biological functions to them. The next stage may instead focus on understanding how these molecules interact within organized metabolic systems whose collective behavior cannot be inferred from any single metabolite alone. In this view, biological information resides not only in molecular structure but also in the architecture, dynamics and connectivity of sterol metabolism.

Two centuries after Chevreul isolated and named *cholestérine*, cholesterol itself no longer appears to be the endpoint of the story. Instead, it marks the entry into an increasingly complex metabolic landscape in which biosynthetic sterols, oxysterols and epoxycholestanoids are continuously redistributed to generate new biological functions. The central question for the coming years may therefore no longer be "What does this sterol do?" but rather "How does the sterol network organize, integrate and transmit biological information?" Answering that question may ultimately redefine not only sterol biology but our broader understanding of metabolic regulation itself.

## Conclusion

The history of sterol biology illustrates how advances in experimental methods have repeatedly transformed our understanding of cholesterol metabolism. Chevreul first established cholesterol as a distinct chemical entity, Bloch revealed the remarkable evolutionary logic underlying its biosynthesis, and Brown and Goldstein uncovered the regulatory circuits that maintain sterol homeostasis (1, 3, 4, 14). Each of these conceptual advances expanded the biological significance of cholesterol beyond what had previously been imagined.

The evidence reviewed here suggests that another transition is now underway. Cholesterol biosynthesis can no longer be viewed solely as a pathway that culminates in cholesterol production. Instead, it emerges as a dynamic metabolic system capable of continuously generating, redistributing and transforming structurally related sterols into functionally distinct metabolic states. In this perspective, biosynthetic intermediates, oxysterols and epoxycholestanoids are not independent metabolic curiosities but interconnected components of a highly organized sterol network.

This view helps reconcile many observations that previously appeared unrelated. Developmental disorders, pharmacological perturbations, cancer, immune responses, neuroregeneration and ageing all reveal that relatively small changes in sterol composition can profoundly influence membrane organization, intracellular signaling, oxidative metabolism and cell fate. The common denominator is not cholesterol itself, but the way sterol metabolism is dynamically organized.

Rather than diminishing the importance of cholesterol, this framework places it within its broader biological context. Cholesterol remains the central structural sterol of mammalian cells, but its biological functions cannot be fully understood independently of the metabolic network from which it arises and the diverse sterol metabolites that accompany it. The pathway is therefore defined not only by its final product but also by the organization of the metabolic landscape that it continuously creates.

Future progress will undoubtedly reveal additional sterol-derived signaling molecules, new enzymatic pathways and previously unrecognized metabolic connections. Yet perhaps the most important challenge will be conceptual rather than analytical: understanding how the organization of sterol metabolism itself contributes to biological regulation.

Two centuries after Chevreul first isolated *cholestérine*, and more than 40 years after Bloch proposed cholesterol biosynthesis as an evolutionary process of molecular optimization, sterol biology appears ready for another conceptual step. The next frontier may no longer be the discovery of individual sterol molecules, but the understanding of how sterol networks are assembled, remodeled and exploited by cells to coordinate physiology and disease. In that sense, cholesterol biosynthesis is not simply a pathway that produces cholesterol, it is an adaptive metabolic system whose organization generates biological information.

## Data availability

All data presented are contained within the manuscript.

## Conflict of interest

The authors declare that they have no conflicts of interest with the contents of this article.
